# Effects of Guideline-Based Correction of Platelet Inhibition on Outcomes in Moderate to Severe Isolated Blunt Traumatic Brain Injury

**DOI:** 10.1089/neur.2022.0003

**Published:** 2022-09-22

**Authors:** Andrew B. Sorah, Kyle Cunningham, Huaping Wang, Colleen Karvetski, Michael Ekaney, Rita Brintzenhoff, Susan Evans

**Affiliations:** ^1^ F.H. Sammy Ross Trauma Center, Carolinas Medical Center, Charlotte, North Carolina, USA.; ^2^ Information and Analytic Services, Carolinas Medical Center, Charlotte, North Carolina, USA.

**Keywords:** platelet dysfunction, platelet transfusion, thromboelastography, traumatic brain injury

## Abstract

Platelet dysfunction has been demonstrated after traumatic brain injury (TBI) regardless of the use of platelet inhibitors. The purpose of this study was to determine the efficacy of a platelet-mapping thromboelastography (PM-TEG) in predicting TBI patients who would benefit from platelet transfusion. We hypothesized that adenosine diphosphate (ADP) and arachadonic acid (AA) inhibition in patients with TBI is associated with increased mortality and can be corrected with platelet transfusion. This is a retrospective review of patients admitted to a level 1 trauma center from January 2016 through September 2017 with moderate to severe blunt TBI (msTBI), defined by an initial Glasgow Coma Scale (GCS) ≤12 with intracranial hemorrhage. Patients received PM-TEG. Those with platelet dysfunction (ADP or AA inhibition ≥60%) received one unit of platelets followed by repeat PM-TEG, until inhibition <60% or three units of platelets. Cohorts were defined as patients initially without (NPI) and with (PI) inhibition and subdivided into those whose inhibition corrected (PI-C) versus those whose did not correct (PI-NC). From 69 patients with isolated blunt TBI, 40 (58%) presented with NPI, 29 (42%) with PI. Of those with PI, 16 (55%) were with PI-C and 13 (45%) with PI-NC. Platelet inhibition in msTBI patients undergoing guideline-based transfusion is associated with age and GCS and an increase in mortality. Platelet inhibition seems to have a more adverse effect on patients >55 years of age or with GCS <8. Correction of platelet inhibition normalized mortality to that of NPI.

## Introduction

Coagulopathy attributable to platelet dysfunction is a common occurrence in patients with moderate to severe (msTBI) traumatic brain injury (TBI) and is associated with poor outcome.^1–3^ Specifically, platelet inhibition at the adenosine diphosphate (ADP) receptor is independently associated with in-hospital mortality in patients with TBI.^1^ Further, ADP inhibition has been shown to be linked to higher mortality in severe TBI.^2^ Platelet dysfunction has been identified in msTBI patients regardless of pre-injury use of platelet inhibitors, platelet count, or coagulation factors on standard laboratory testing.^3–5^ As a result, routine screening of msTBI patients with coagulation panels and platelet counts alone will not dependably identify the patient at risk of further bleeding.

Platelet transfusion is currently the only routine therapy available to correct platelet dysfunction. The benefit of platelet transfusion in patients with msTBI remains controversial. In a large retrospective analysis of trauma patients, the need for transfusion was not predicted by platelet ADP inhibition.^3^ Platelet transfusion in traumatic injuries has primarily been utilized for patients who take platelet inhibitor therapy. In several studies, patients with TBI received platelet transfusion at the discretion of the managing physician if the patient had a known history of platelet inhibitor use.^4,6–8^ These studies concluded that there was no difference in outcome after transfusion; however, the patients who received transfusions had markers of greater severity of injury that would have typically predicted worse outcomes. This suggests that the transfusions may have been effective in improving outcomes of the more severely injured patients.

The purpose of this study was to review the application of a platelet transfusion guideline and identify its effects on platelet dysfunction of moderate and severe isolated blunt TBI patients. Platelet dysfunction, and the need for transfusion, was determined by platelet-mapping thromboelastography (PM-TEG) in the guideline. We hypothesized that guideline-based platelet transfusion would result in corrected adenosine diphosphate (ADP) and arachidonic acid (AA) inhibition and that correction would be associated with improved patient outcomes. These findings could then be used to inform the design of a prospective, controlled study to determine platelet transfusion efficacy in moderate and severe isolated blunt TBI patients.

## Methods

### Setting

This retrospective study of the implementation of an msTBI platelet transfusion guideline was conducted at Carolinas Medical Center, a level 1 trauma center in Charlotte, North Carolina. Patients who were treated by the msTBI guideline between January 2016 and September 2017 with a blunt injury mechanism and isolated TBI were included in the medical record review. The study was approved by the Carolinas Medical Center Institutional Review Board. Patients were excluded for penetrating mechanism of injury or multi-system trauma, defined by Abbreviated Injury Scale (AIS) ≥3 in any body region other than the head.

### Platelet transfusion guideline

The platelet transfusion guideline was developed to treat msTBI patients who presented with severe intracranial bleeding and demonstrated platelet inhibition based on PM-TEG measurement. Platelet dysfunction was defined as inhibition ≥60% in the AA or ADP pathway based on PM-TEG. This value of platelet inhibition was selected because it is considered appropriately anticoagulated in patients treated with platelet inhibitors.^9^

**FIG. 1.**
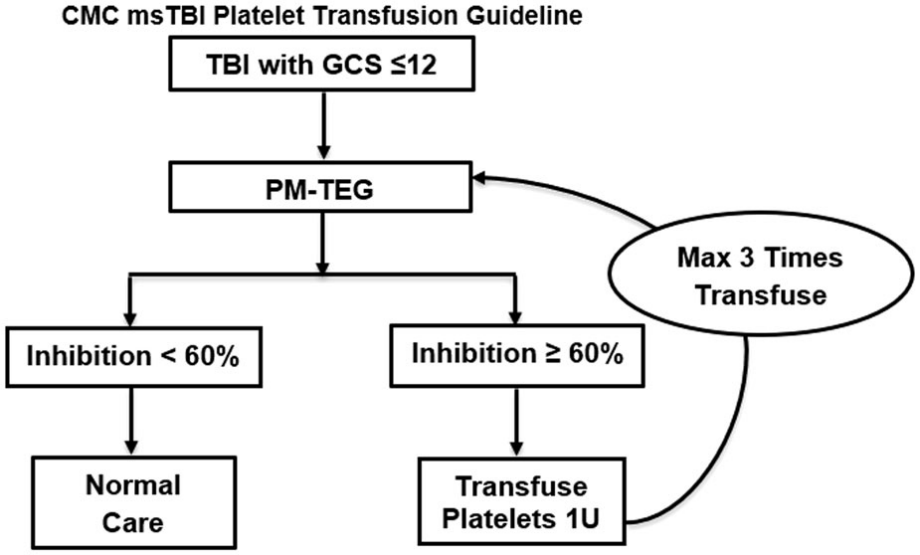
Algorithm of Initial Management of msTBI. Patients who have GCS <13 and blood on CT receive platelet-mapping thromboelastograhy. Those who demonstrate either adenosine diphosphate (ADP) or arachadonic acid (AA) inhibition >60% receive a transfusion of one unit of platelets. This repeats until inhibition is <60% or three transfusions have occurred. All other care is directed by the normal practice of the trauma service. CMC, Carolinas Medical Center; CT, computed tomography; GCS, Glasgow Coma Scale; msTBI, moderate to severe TBI; PM-TEG, platelet-mapping thromboelastography; TBI, traumatic brain injury.

Patients ≥18 years of age who presented with msTBI were evaluated for application of the platelet transfusion guideline. Among those, any patient who also had a Glasgow Coma Scale (GCS) score ≤12 and intracranial hemorrhage detected by initial head computed tomography (CT) were appropriate for the guideline. An initial PM-TEG was obtained, per guideline, either upon arrival to the surgical trauma intensive care unit or to the operating room. Patients who demonstrated platelet dysfunction were administered one unit of platelets. A PM-TEG was then repeated at 1 h post-transfusion. If inhibition persisted, another platelet transfusion was administered. Platelet transfusion continued until a maximum of three units of platelets were transfused or until PM-TEG demonstrated no inhibition (inhibition <60%). Patients with multi-system polytrauma were included in our institutional treatment guidelines. However, only those with isolated msTBI were included in this study (Fig. 1).

### Statistical analysis

Patients were separated into two groups based on baseline platelet inhibition status: patients without (NPI) and with (PI) platelet inhibition. Initial analyses were performed between these two groups. To further investigate the impact of platelet correction, those with PI were further divided into two groups: patients in whom inhibition corrected (PI-C) and did not correct after receiving platelet transfusion (PI-NC). Outcomes between PI-C, PI-NC, and NPI were compared while adjusting for age and GCS in multi-variate statistical models. Subsequent analyses were performed after dividing patients by age ≤55 (younger) and >55 (older) and GCS into ≤8 (severe TBI) with 9–12 (moderate TBI) when evaluating interaction effects.

The primary outcome measure was hospital mortality from all causes. Additional outcome measures included days of mechanical ventilation, length of intensive care unit (ICU) stay, and length of hospital stay. Age, sex, GCS, AIS scores, and thrombin maximum amplitude (MA) were obtained from the electronic medical record or trauma database registry.

All data were entered into the Research Electronic Data Capture (REDCap) database. Two-tailed *p* values were calculated for all tests, and *p* < 0.05 was considered statistically significant. For continuous variables, we used general linear regression models if their distributions met normality assumptions. Otherwise, we used robust regression models. For binary variables, we used logistic regression models or chi-square tests. We report mean and standard deviation (SD) and median and interquartile range (IQR) for normally and non-normally distributed continuous variables, respectively. We presented odds ratio (OR) and 95% confidence interval (CI) for mortality and regression coefficients and 95% CI for other outcome variables. SAS software (version 9.4; SAS Institute Inc., Cary, NC) was used for all data analysis.

## Results

### General patient characteristics and inhibition

**FIG. 2.**
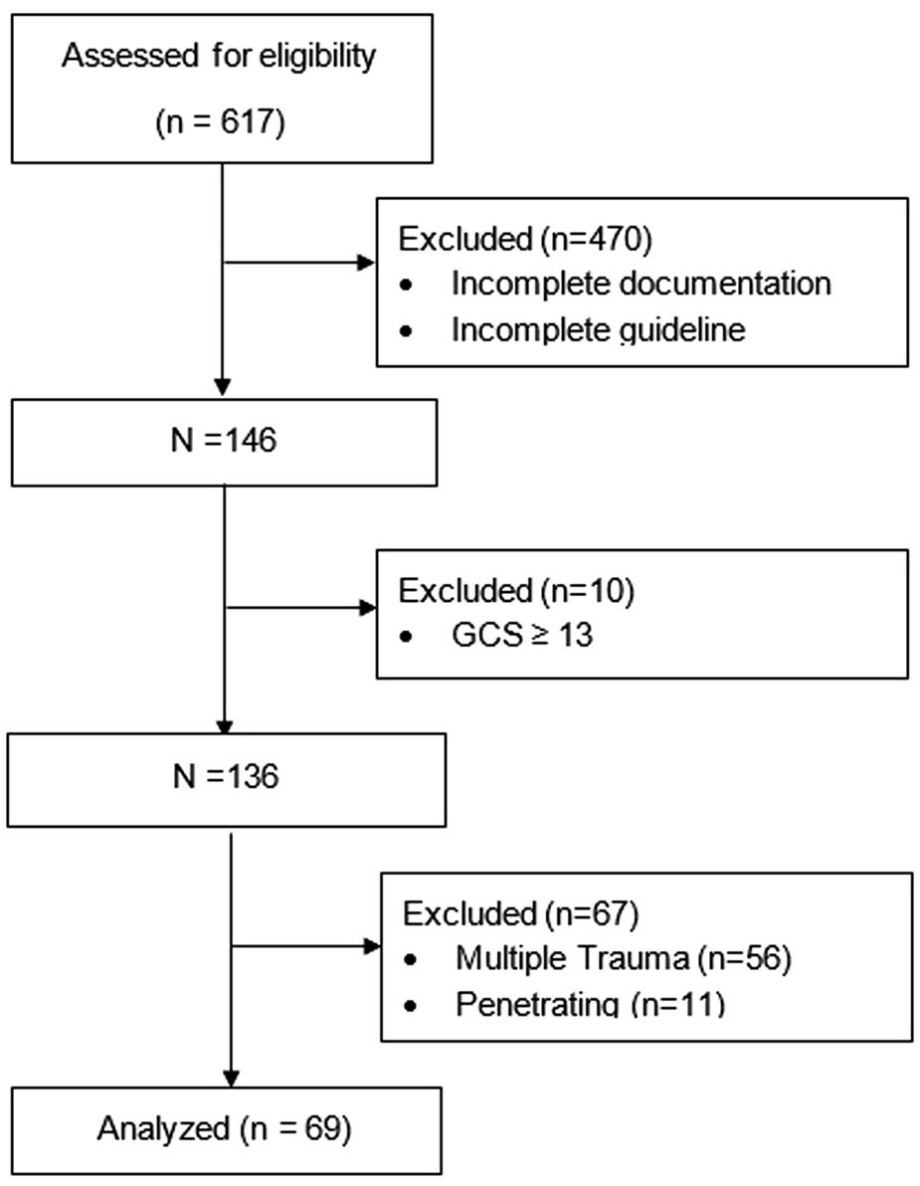
Inclusion and exclusion of patients. GCS, Glasgow Coma Scale.

Of the 146 patients eligible for the study based on completion of the guideline, 10 patients were excluded for GCS ≥13, 56 were excluded for multi-system trauma, and 11 were excluded for penetrating injury mechanism. Figure 2 outlines the eligible population. The remaining 69 patients were the subjects of the current analysis (see Fig. 2). Forty-five (65.2%) of the 69 patients in this study were male. Average age was 51.3 (±18.7) years, and average GCS was 7.6 (±2.6). All groups had similar AIS Head scores as well as similar baseline characteristics, and no platelet transfusion reactions were observed in the included patients.

Table 1 presents 69 patients' baseline characteristics for each group. Forty (58%) patients showed no platelet inhibition (NPI), and 29 (42%) patients showed platelet inhibition (PI). Among the 29 patients, 16 had correction (PI-C) and 13 did not have correction (PI-NC). Compared to NPI patients, PI patients were similar in age, sex, GCS, and thrombin MA. Mortality was not different between the groups on single-variable regression analysis (OR [95% CI], 1.9) but when corrected for age and GCS the OR for mortality was 4.8 (95% CI [1.01, 18.43]) for PI (Table 2).

**Table 1.** Characteristics of 69 Isolated Blunt Patients

	NPI	PI		PI-C	PI-NC	
	*n* = 40	*n* = 29	*p* value^*^	*n* = 16	*n* = 13	*p* value^**^
Age, years, mean (SD)	52.4 (18)	49.9 (19.9)	0.586	46.5 (21)	54 (18.3)	0.32
Sex, *n* (%)			0.285			1
Female	16 (40)	8 (27.6)		4 (25)	4 (30.8)	
Male	24 (60)	21 (72.4)		12 (75)	9 (69.2)	
AIS_Head, median (IQR)	4 (3–5)	4 (4–5)	0.75	4 (3–5)	4 (4–4)	0.78
Glasgow Coma Scale, mean (SD)	7.6 (2.5)	7.6 (2.8)	0.97	8.0 (2.9)	7.2 (2.8)	0.43
Injury Severity Score, median (IQR)	22.5 (15–26)	24 (17–26)	0.77	22.5 (12–26)	24 (17-24)	0.84
Thrombin maximum, median (IQR)	63 (61–66)	63 (58–66)	0.69	63 (63–67)	62 (51–64)	0.16
Percent ADP base, median (IQR)	31.7 (12.0–44.4)	97 (88–100)	<0.0001	97 (88–100)	99 (89.2–99.0)	0.98
Percent AA base, median (IQR)	18 (5.0–31.5)	67 (34–99)	<0.0001	45.7 (33.6–72.9)	94 (62–100)	0.054

^*^ *p* value between NPI and PI.

^**^ *p* value between PI-C and PI-NC.

AA, arachadonic acid; ADP, adenosine diphosphate; AIS, Abbreviated Injury Scale; IQR, interquartile range; SD, standard deviation.

**Table 2.** Comparing Outcome between PI and NPI

Outcome measure	NPI	PI	Unadjusted results		Adjusted results	
	*n* = 40	*n* = 29	OR (95% CI)	*p* value	OR (95% CI)	*p* value
Mortality, *n* (%)	11 (27.5)	12 (41.4)	1.9 (0.67, 5.13)	0.230	4.3 (1.01, 18.43)	0.0480
Age					1.1 (1.04, 1.14)	0.0004
Glasgow Coma Scale					0.5 (0.38, 0.76)	0.0005

			β (95% CI)	*p* value	β (95% CI)	*p* value
Ventilator, days, median (IQR)	3.3 (1.7–9.8)	4.3 (1.2–7.8)	–0.3 (−2.56, 2.02)	0.817	–0.3 (−2.76, 2.09)	0.7863
Age					–0.02 (−0.09, 0.05)	0.5247
Glasgow Coma Scale					–0.1 (−0.55, 0.40)	0.7571
ICU, days, median (IQR)	5.5 (2.2–10.1)	4.8 (1.7–7.6)	–0.5 (−2.8, 1.9)	0.707	–0.5 (−2.84, 1.81)	0.6655
Age					–0.03 (−0.09, 0.03)	0.3467
Glasgow Coma Scale					–0.1 (−0.54, 0.34)	0.6535
Hospital stay, days, median (IQR)	12 (8–18)	14 (4–20)	0.3 (−4.71, 5.31)	0.907	0.2 (−4.71, 5.17)	0.9281
Age					–0.1 (−0.27, −0.00)	0.0429
Glasgow Coma Scale					0.8 (−0.10, 1.79)	0.0797

ICU, intensive care unit; IQR, interquartile range; OR, odds ratio; CI, confidence interval.

### Correction of inhibition on outcome

To further determine the influence of correction of platelet inhibition on outcomes, we compared PI-C, PI-NC, and NPI in patients' outcomes. Results are presented in Table 3. The PI-NC group had the highest mortality rate (61.5%). Univariate analysis showed that patients whose platelets did not correct (PI-NC) were 4.8 times more likely to die (95% CI [0.98, 23.54], *p* = 0.0532) compared to those who corrected (PI-C) and 4.2 times more likely to die (95% CI [1.13, 15.71], *p* = 0.0320) compared to those who did not show platelet inhibition (NPI). However, the difference between PI-NC and PI-C was no longer observed after correction for both age and GCS. Additionally, age and GCS at baseline were found to be associated with increased mortality. (Multi-variate analysis showed a 10% increased risk of death per year of increase in age (OR [95% CI], 1.1 [1.0, 1.2]; *p* = 0.0005) and a 50% decreased risk of death per unit increase in GCS (OR [95% CI], 0.5 [0.4, 0.8]; *p* = 0.0006).) However, both unadjusted and adjusted results indicated no differences in ventilator days and ICU length of stay (LOS) among the three groups, but advanced age was associated with greater hospital LOS in each group.

**Table 3.** Platelet Correction Effects

	NPI	PI-C	PI-NC		Unadjusted		Adjusted	
	*n* = 40	*n* = 16	*n* = 13		OR (95% CI)	*p* value	OR (95% CI)	*p* value
Mortality, *n* (%)	11 (27.5)	4 (25)	8 (61.5)	PI-NC vs. PI-C	4.8 (0.98, 23.54)	0.0532	4.2 (0.51, 34.51)	0.1838
				PI-C vs. NPI	0.9 (0.23, 3.31)	0.8487	1.8 (0.26, 12.40)	0.5497
				PI-NC vs. NPI	4.2 (1.13, 15.71)	0.0320	7.5 (1.41, 40.14)	0.0180
Age							1.1 (1.04, 1.15)	0.0005
Glasgow Coma Scale							0.5 (0.39, 0.77)	0.0006

					β (95% CI)	*p* value	β (95% CI)	*p* value
Ventilator, days, median (IQR)	3.3 (1.7–9.8)	3.7 (0.6–7.5)	4.5 (2.2–8.3)	PI-NC vs. PI-C	0.7 (−2.74, 4.23)	0.6738	0.9 (−2.81, 4.56)	0.6409
			2 vs. 3	PI-C vs. NPI	–0.6 (−3.43, 2.17)	0.6595	–0.8 (−3.73, 2.21)	0.6175
			1 vs. 3	PI-NC vs. NPI	0.1 (−2.84, 3.07)	0.9376	0.1 (−2.93, 3.72)	0.9388
Age							–0.02 (−0.09, 0.04)	0.4852
Glasgow Coma Scale							–0.1 (−0.53, 0.41)	0.7967
ICU, days, median (IQR)	5.5 (2.2–10.1)	4.3 (1.6–7.5)	6.2 (2.6–9.9)	PI-NC vs. PI-C	1.6 (−1.89, 5.10)	0.3684	1.7 (−1.89, 5.31)	0.3518
			2 vs. 3	PI-C vs. NPI	–1.2 (−3.96, 1.57)	0.3981	–1.3 (−4.14, 1.54)	0.3706
			1 vs. 3	PI-NC vs. NPI	0.4 (−2.58, 3.40)	0.7879	0.4 (−2.63, 3.45)	0.7897
Age							–0.03 (−0.10, 0.03)	0.3049
Glasgow Coma Scale							–0.1 (−0.52, 0.37)	0.7420
Hospital stay, days, median (IQR)	12 (8–18)	14 (4–24)	10 (4–20)	PI-NC vs. PI-C	–1.9 (−9.64, 5.80)	0.6261	–0.2 (−8.02, 7.63)	0.9608
			2 vs. 3	PI-C vs. NPI	1.2 (−4.87, 7.37)	0.6893	0.3 (−5.83, 6.50)	0.9154
			1 vs. 3	PI-NC vs. NPI	–0.7 (−7.27, 5.93)	0.8419	0.1 (−6.46, 6.74)	0.9673
Age							–0.1 (−0.27, −0.00)	0.0498
Glasgow Coma Scale							0.8 (−0.13, 1.81)	0.0905

IQR, interquartile range; OR, odds ratio; CI, confidence interval.

## Discussion

The mortality rate of patients with msTBI remains high. Our study demonstrates that patients with uncorrected platelet inhibition have a 4-fold greater OR of mortality compared to those without platelet inhibition after correction for age and GCS. Interestingly, those patients who present with platelet inhibition, yet have correction of that inhibition with platelet transfusion, have similar mortality to those who were never inhibited. It is impossible to determine from this study whether this is because the platelet transfusion improved mortality risk or whether the ability to correct inhibition is a biomarker of outcome.

Our findings are similar to other studies demonstrating that baseline platelet ADP inhibition >60% in TBI patients is associated with a higher mortality rate.^1,3,10^ Some studies have not found a similar relationship between platelet inhibition and outcome in TBI in the setting of platelet-inhibiting medications.^11,12^ The differences may be attributable to the practice of transfusing some of the patients by physician discretion, thus introducing treatment bias. If the patients selected for transfusion had a higher chance of responding to the transfusion with correction of inhibition, they could be expected to display similar mortality to those who presented without inhibition. Our findings would support such a hypothesis. It would be helpful to determine whether the outcome of inhibited patients would have been worse without the transfusion—a question that can only be definitively answered with randomization of inhibited patients to transfusion and no transfusion.

We also found that patients with persistent platelet inhibition despite platelet transfusion have a nearly 8-fold increased odds of mortality compared to patients without inhibition at baseline even after correction for GCS and age. The relationship between platelet inhibition and mortality in TBI certainly warrants additional investigation. Inability to correct inhibition appears to function as a biomarker of poor outcome at a minimum. Unfortunately, it is impossible to delineate at the time of presentation which patients will correct and those who will not.

### Relationship of age and inhibition on mortality

**FIG. 3.**
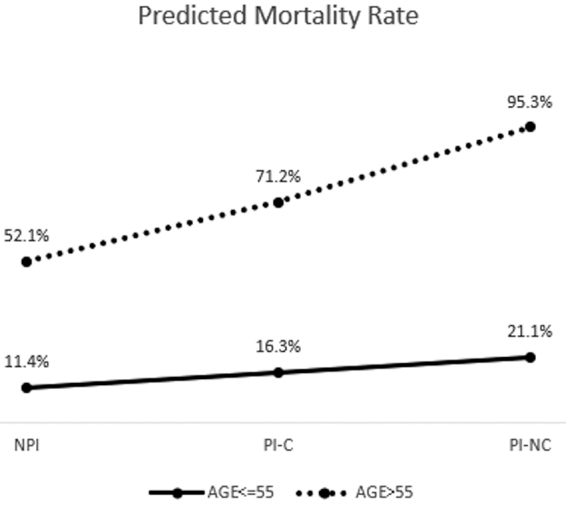
Interaction effects by age.

In our clinical experience, we observed very pronounced inhibition in the youngest patients, and because age was highly associated with outcome in the model, we wanted to better understand this relationship. Therefore, we dichotomized age into two groups: ≤55 and >55 and compared groups on predicted mortality rates (Fig. 3) from a multi-variate logistic regression model. This revealed small differences in mortality between younger patients, when stratified by inhibition status, and substantial differences in mortality between inhibition groups in the older patient population. The difference is most pronounced in the PI-NC group between those <55 and those >55 years of age. Older patients with higher levels of platelet inhibition demonstrate markedly increased mortality.

### Relationship of Glasgow Coma Scale and inhibition on mortality

**FIG. 4.**
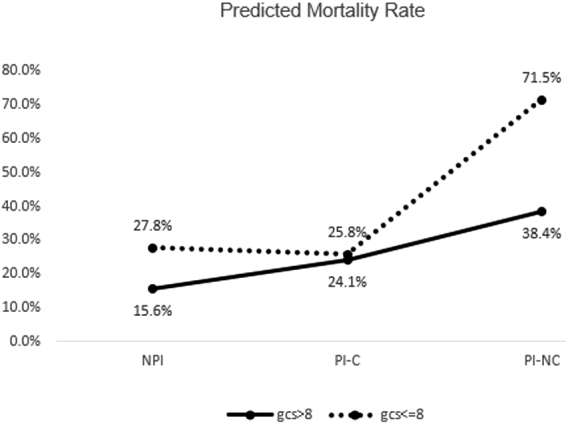
Interaction effects by Glasgow Coma Scale.

Similar to age, we wanted to determine whether there was an interaction between GCS and inhibition group with mortality (Fig. 4). The mortality risk for patients with moderate TBI was somewhat increased in patients whose platelet inhibition did not correct. However, for severe TBI, those whose inhibition did not correct had a substantially higher mortality than those whose inhibition corrected after transfusion. This may suggest that the benefit of transfusion is most notable in those with the most severe injury. Mortality was greatly increased for inhibited patients whose platelet inhibition was not corrected, compared to patients whose inhibition corrected (71.5% vs. 38.4%).

Previous studies have demonstrated a relationship between platelet ADP inhibition and poor outcome in humans^1,2^ and animal models.^13^ A recent study also shows promising results of transfusion in patients with severe TBI and platelet dysfunction. Patients who were transfused with a similar guideline (although ceasing at two units of platelets) had a trend toward decreased mortality, which did not reach significance after controlling for age, GCS, and Injury Severity Score (ISS).^7^ However, others have not identified a similar relationship. In a large group of minimally injured patients (median ISS = 5), ADP inhibition did not correlate with ISS, LOS, or mortality, even in the subgroup of TBI patients. ADP inhibition did differ between patients with ISS >15 and <15 in TBI patients.^11^

The lack of a direct association between inhibition and mortality is discrepant from our findings. This may be related to the generally low ISS in this group, which is also associated with lower mortality. In a group of injured patients, both minimally and severely injured, ADP inhibition did not provide additional value over thrombin-induced maximal clot strength on rapid TEG to mortality predictions.^14^ This was in contrast to our findings. However, the quoted study included patients with multi-system trauma, both blunt and penetrating. In that subset of the patient population with likely large-volume blood loss, ADP inhibition may not add significantly to the overall clinical picture. However, in patients with isolated TBI and the known related platelet dysfunction, without large-volume hemorrhage resulting in depletion and derangement of many components of the coagulation cascade, ADP inhibition likely adds significantly to the prognostic ability of the treating physician.

The association between platelet dysfunction and patient outcome has at least two plausible explanations. Poor clot strength would predispose a patient to ongoing or repeat bleeding, either intracranially or in a remote site of injury, resulting in higher morbidity and mortality. Alternatively, the greater platelet inhibition could simply be a biomarker of higher injury burden and altered physiology. Before this study, it was not certain whether this platelet dysfunction, as it relates to ADP inhibition, is merely prognostic or may be a target for therapeutic intervention with platelet transfusion, or another therapy not yet determined. In our data set, it does appear that platelet transfusion, when given to the appropriate subset of patients, can lead to a decreased mortality rate if correction of platelet inhibition can be achieved. Other studies have demonstrated no mortality benefit of transfusion when evaluating patients taking platelet inhibitors.^6,8,14^ However, in those studies, the patients receiving transfusions had more extensive intracranial hemorrhage and a higher predicted mortality. Therefore, equal mortality suggested a possible benefit of transfusion.

The pathophysiology behind the observed platelet dysfunction is likely multi-factorial, and the exact etiology remains elusive. A “platelet exhaustion syndrome” has been previously hypothesized resulting in release of brain tissue factor with subsequent activation of factor VIIa, systemic thrombin production, and platelet activation.^15,16^ Microparticle procoagulant release and activity have also been implicated in TBI-induced platelet dysfunction, leading to overall decreased maximum clot firmness.^17^ These microparticles have been identified in both cerebrospinal fluid and peripheral blood where they contribute to disseminated intravascular coagulation in some patients through an overwhelming thrombus inhibition and antithrombin consumption.^18^

Previous studies have shown improvement in platelet function after transfusion in TBI patients taking aspirin, but did not show improvement with transfusion of a single unit of apheresis platelets in those with TBI-induced platelet dysfunction not on aspirin.^18^ However, these patients may have had subsequent improvement in their dysfunction had they received more than one unit of platelets during resuscitation. Because platelets are a limited resource, judicious use should be practiced; a guideline which provides the mechanism to determine when to proceed with transfusion based on a physiological parameter can assist with appropriate, effective transfusion practices and use of system resources.

The question remains, “Why did some patients not have successful correction of inhibition?” Age and baseline thrombin MA were not different between those with and without successful correction of inhibition, suggesting that these do not explain lack of correction. It is possible that the quality of transfused platelets could be different between these groups. Patients who did not correct may have received stored platelets with lower aggregation. Age of infused platelets was not evaluated in this study. To date, the influence of stored platelet function has not been determined.

We did not discriminate between patients who had taken platelet inhibitor or anticoagulant medications and those who did not. It has been demonstrated that up to 41% of patients on platelet inhibitors demonstrate no platelet inhibition.^9,19^ In crafting our practice guideline *a priori*, we decided that assessing platelet function was more relevant than using history of platelet inhibitor medications. Additionally, baseline inhibition in healthy humans not taking platelet inhibitors can range from 0% to 58%.^20^ Consequently, the extent of inhibition was more important to patient management than historical information.

A limitation of the study is that the algorithm does require the use of PM-TEG. This is not available at all facilities and could be a limiting factor at some institutions serving as the initial resuscitative team for patients with TBI. Also, our sample was small, which limited our study power. The retrospective design and chart review based on an algorithm-based protocol introduces additional limitations. Further studies with a randomized controlled design would greatly benefit outcome interpretation.

Future work to investigate platelet dysfunction attributable to platelet-inhibiting medications and end-points other than in-hospital mortality—such as progression of intracranial hemorrhage, requirement of operative intervention, and functional outcomes—would provide substantial insight into correction of platelet function. Comparing patients with isolated TBI and multi-system polytrauma would also offer guidance for transfusion in varied patient populations.

## Conclusion

Platelet inhibition in moderate and severe isolated msTBI is associated with increased mortality, and correction of platelet inhibition subsequent to guideline-based transfusion is associated with normalization of mortality to that of patients without inhibition. Further, lack of correction of platelet inhibition with platelet transfusion in msTBI is an amplified marker of mortality and most pronounced in patients >55 years of age. Practices that include guideline-based assessment of platelet function to direct transfusion may be beneficial in resource allocation. Additional study of the mechanisms involved will help elucidate prognosis and define patients who could benefit from platelet transfusion post-TBI.

## Abbreviations Used

AA: arachadonic acid
ADP: adenosine diphosphate
AIS: Abbreviated Injury Scale
CI: confidence interval
CT: computed tomography
GCS: Glasgow Coma Scale
ICU: intensive care unit
IQR: interquartile range
ISS: Injury Severity Score
LOS: length of stay
MA: maximum amplitude
msTBI: moderate to severe traumatic brain injury
OR: odds ratio
PM-TEG: platelet-mapping thromboelastography
SD: standard deviation
TBI: traumatic brain injury
